# Empathy and compassion toward other species decrease with evolutionary divergence time

**DOI:** 10.1038/s41598-019-56006-9

**Published:** 2019-12-20

**Authors:** Aurélien Miralles, Michel Raymond, Guillaume Lecointre

**Affiliations:** 1 Institut de Systématique, Evolution, Biodiversité, (UMR 7205 Muséum national d’Histoire naturelle, CNRS UPMC EPHE, Sorbonne Universités), CP30, 25 rue Cuvier 75005, Paris, France; 2 ISEM, Univ Montpellier, CNRS, EPHE, IRD, Montpellier, France

**Keywords:** Human behaviour, Phylogenetics

## Abstract

Currently the planet is inhabited by several millions of extremely diversified species. Not all of them arouse emotions of the same nature or intensity in humans. Little is known about the extent of our affective responses toward them and the factors that may explain these differences. Our online survey involved 3500 raters who had to make choices depending on specific questions designed to either assess their empathic perceptions or their compassionate reactions toward an extended photographic sampling of organisms. Results show a strong negative correlation between empathy scores and the divergence time separating them from us. However, beyond a certain time of divergence, our empathic perceptions stabilize at a minimum level. Compassion scores, although based on less spontaneous choices, remain strongly correlated to empathy scores and time of divergence. The mosaic of features characterizing humans has been acquired gradually over the course of the evolution, and the phylogenetically closer a species is to us, the more it shares common traits with us. Our results could be explained by the fact that many of these traits may arouse sensory biases. These anthropomorphic signals could be able to mobilize cognitive circuitry and to trigger prosocial behaviors usually at work in human relationships.

## Introduction

“*Sympathy beyond the confines of man, that is, humanity to the lower animals, seems to be one of the latest moral acquisitions. (…). This virtue, one of the noblest with which man is endowed, seems to arise incidentally from our sympathies becoming more tender and more widely diffused, until they are extended to all sentient beings*”.Charles Darwin, 1871^1^.

Whether it be for nutrition, recreational and ritual practices, research or wildlife management, man’s interactions with other organisms are countless, complex and go back to the roots of humankind. The nature of these interactions is not restricted to their utilitarian function. They also convey a diversified and ambivalent emotional component, which can resurface with intensity in social debates about animal welfare or nature conservation, and may even lead to radical actions under certain circumstances^2,3^.

Among the numerous species having evolved on Earth, all the different living organisms do not affect humans evenly. This imbalance is so marked that even scientific research on biodiversity or conservation efforts present a significant bias in favor of our societal inclinations for particular taxa^4,5^. Several factors have been advanced to explain these preferences, such as aesthetics, body size or feeling of vulnerability^6–9^. Nevertheless, the emotional perceptions we can feel for a member of a given species seems to be largely related to its ability to arouse anthropomorphic projections (attribution of human traits, emotions, or intentions to non-human entities). Species exhibiting physical, behavioral or cognitive similarities with humans tend to evoke more positive affect than those without^6^, and among the different classes of vertebrates, our empathic responses appears to be more important for taxa that are closely related to us^10–12^.

Ironically, these comparative studies dealing with anthropomorphic perceptions of biodiversity are mainly restricted to mammals, and in a less extent to the other vertebrates. Moreover, mainly undertaken by cognitive or conservation scientists, these questions have received very little interest from an evolutionary biology perspective. To the extent of our knowledge, they have never been addressed using an adequate comparative methodology, i.e. based on a phylogenetic analytic framework. Several basic but fundamental questions are therefore left unanswered about our ability to connect emotionally with other organisms. Does it apply to all living beings or is it limited to a particular perimeter? To what extent does phylogenetic proximity explain our ability to understand their emotions and to express sympathy towards them? Does it decrease linearly with the time of phylogenetic divergence separating them from us, or stepwise, depending on particular level of organization, i.e. corresponding to evolutionary grades? What is the nature of the stimuli at the origin of these perceptions and how can they arouse in us emotions comparable to those usually expressed within human relationships? And in a broader extent, how can we explain in the frame of the natural selection paradigm, the existence of altruistic behaviors between different species?

**Figure 1:**
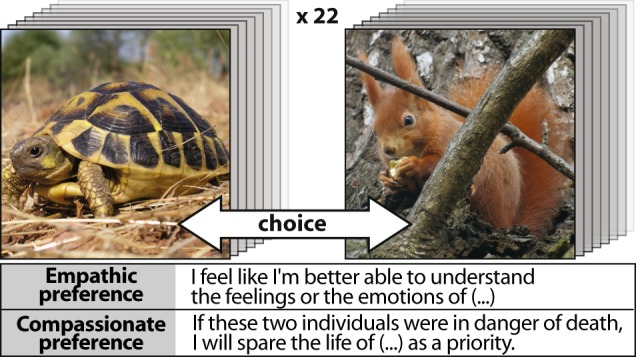
Experimental procedure. Based on a focused question, each evaluator had 22 pairs of pictures to evaluate (randomly drawn from a total of 52 species). The question, also randomly drawn at the beginning of the test, was intended to assess either empathic or compassionate preferences. (photos by A. Miralles).

In order to fill some of these gaps, the present investigatory project was designed to provide the first cartography of the living world through human empathy-related responsiveness it may arouse, and to interpret its variations in a phylogenetic comparative framework. Our online survey involved 3500 raters who had to make preference choices over an extended photographic sampling of organisms, designed to be as representative as possible of the phylogenetic diversity of life (microscopic organisms excluded). Choices were driven by two different questions. Indeed, as there are many different definitions – and a nebulous usage – of the term *empathy*, and a wide array of mental states and notions related to this concept (ex. sympathy, cognitive or affective empathy, compassion, self-other distinction, affect sharing or emotional contagion^13–16^), two different questions were formulated in order to distinguish empathic-like perceptions from compassionate-like responses. The notion of *empathy* is presently referring to the capability to connect with one another at an emotional level^14,17^. The driven question proposed to the raters to assess their empathic preferences was “*I feel like I’m better able to understand the feelings or the emotions of* [choice among a pair of pictures representing different organisms]”. In contrast, the notion of *compassion* (also termed empathic concern) has been used here to refer to the feeling of concern for the suffering of others, associated with a motivation to help^13,18,19^. The corresponding question proposed to raters was “*If these two individuals were in danger of death, I will spare the life of* [choice among a pair of pictures] *as a priority*” (Fig. 1).

## Results

**Figure 2:**
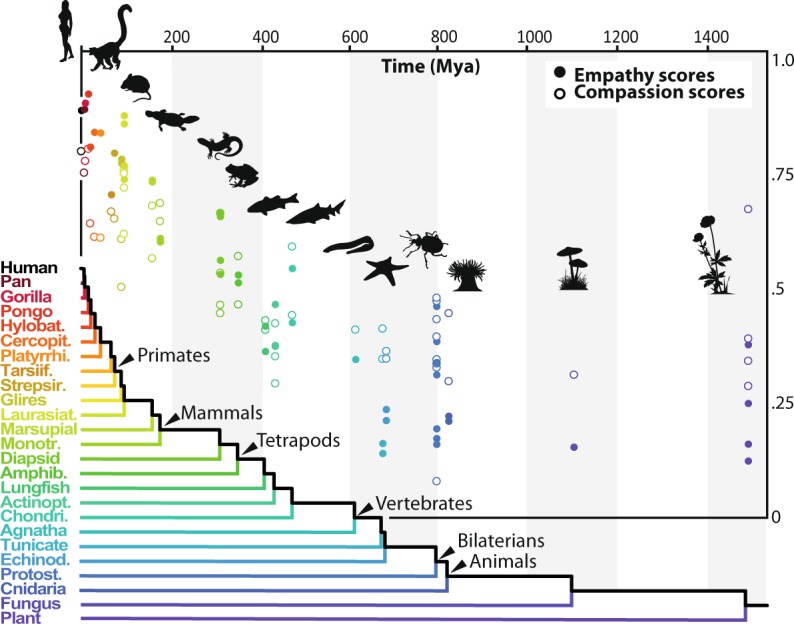
Empathy and compassion scores attributed to each organisms as a function of divergence time (Mya) between them and humans. The scores correspond to the probability that a given species is chosen from a pair of species that includes it and another randomly selected (n = 52 species). See *SI Appendix, Results S1* for details. (Illustrations by A. Miralles).

For the question related to empathy, the probability to be chosen decreased with the phylogenetic distance relatively to humans, compared to the alternative species (Fig. 2, *SI Appendix*, Tables S1 to S4, Figure S1).

**Figure 3:**
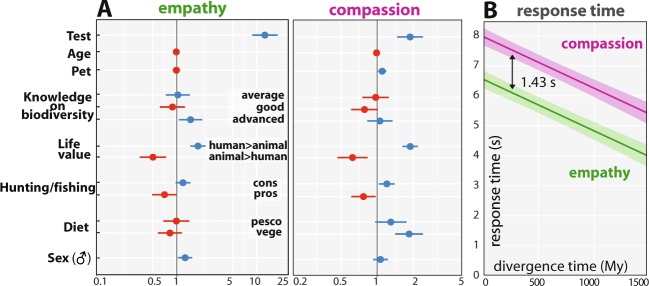
Effect of confounding variables and response time. (**A**) Effect of rater’s traits on both questions. Odds ratio (for a qualitative variable: ratio of the odds of choosing the most phylogenetically related species in the depicted factor level to the odds of it occurring in the reference factor level; for age, centered variable: ratio of the odds of choosing the most phylogenetically related species in age 1 to the odds of it occurring in age 0) are represented by dots and 95% confidence interval by lines; blue or red dots indicate variables linked with an increased or decreased, respectively, choice probability for the most phylogenetically related species (n raters = 1134 for the empathy test and 1213 for the compassion test). (**B**) Predicted participants’ response time as a function of the absolute divergence time between the two species presented in each pair (area depicts the 95% confidence interval, n responses = 25001 for the empathy test and 26781 for the compassion test).

For each relative reduction of phylogenetic distance of one million year, the probability to be chosen increased by 2.54 (SE = 0.19) in linear units (logit). Results varied according to the raters’ sex (P = 0.02), age (P < 0.001), knowledge on biodiversity (P < 0.001), opinion on hunting and fishing (P = 0.01), and opinion on the value of animal life relatively to humans (P < 0.001). Direction of effects are indicated in Table S3, and depicted in Fig. 3A. The empathy score, computed for each species, varied between 0.12 to 0.91, and decreased quadratically with divergence time (linear slope: −1.2 10^−3^, F_1,49_ = 258, P < 10^−16^; quadratic term: 5.3 10^−7^, F_1,49_ = 99.8, P < 10^−13^). From divergence time higher than 611.1 Mya, the empathy score was no longer decreasing with divergence time (estimated inflexion point, with a 95% confidence interval running from 518 to 703 Mya).

For the question related to compassion, the probability to be chosen decreased with the phylogenetic distance relatively to humans, compared to the alternative species (Fig. 2, *SI Appendix*, Tables S1 to S4, Figures S1). For each relative reduction of phylogenetic distance of one million year, the probability to be chosen increased by 0.63 (SE = 0.13) in linear units (logit). Results varied according to the raters’ age (P < 0.001), diet (P < 0.001), knowledge on biodiversity (P = 0.01), opinion on hunting and fishing (P = 0.001), opinion on the value of animal life relatively to humans (P < 0.001), and number of pets (P = 0.016). Direction of effects are indicated in Table S3, and depicted in Fig. 3A. The compassion score, computed for each species, varied between 0.08 to 0.79, and decreased quadratically with divergence time (linear slope: −7.8 10^−4^, F_1,49_ = 76.6, P < 10^−11^; quadratic term: 3.9 10^−7^, F_1,49_ = 39.0, P < 10^−8^). From divergence time higher than 564.9 Mya, the compassion score was no more decreasing with divergence time (estimated inflexion point, with a 95% confidence interval running from 413 to 797 Mya).

**Figure 4:**
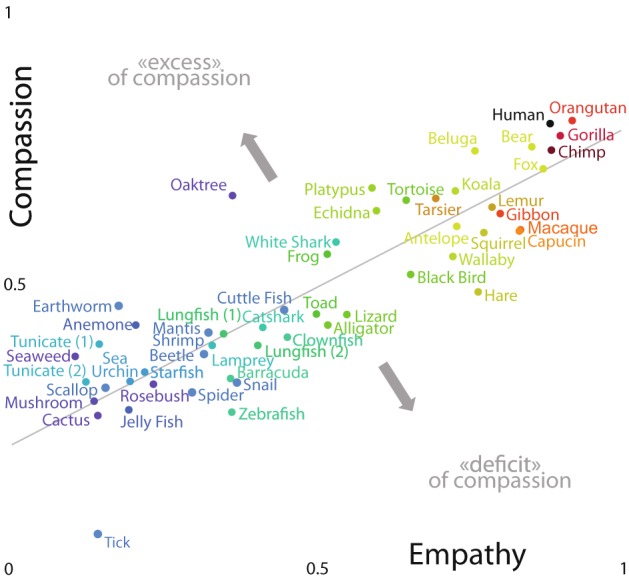
Relationships between empathy and compassion scores. While the oak benefits from an excessive compassion score when compared to its ability to arouse empathy, the tick suffers from a clear compassion deficit (n = 52 species).

The empathy and compassion scores were correlated (Pearson’s product-moment correlation = 0.868, t = 12.4, df = 50, P < 10^−15^). The decrease in score with divergence time was faster for empathic scores than for compassion scores (difference in linear slope: 0.156, SE = 3.24 10^−2^, F_1,98_ = 14.1, P = 3 10^−4^; difference in quadratic terms: F_1,98_ = 2.95, P = 0.089) (Fig. 4, *SI Appendix*, Table S4).

The mean response time of raters decreases significantly with the absolute time of divergence between two organisms, regardless of the question asked (empathy or compassion driven) (Fig. 3B). It decreases by 0.168 s (SE = 0.012) for each increase of divergence time of 100 Myr. When the two species in the pair were equally divergent (absolute time of divergence = 0), mean response time was 7.96 s (SE = 0.15) and 6.54 (SE = 0.15) to empathy and compassion driven questions, respectively. This difference in response time between the two questions, 1.43 s (SE = 0.16), was independent of the absolute divergence time (interaction between absolute divergence time and the type of question: X^2^ = 0.003, df = 1, P = 0.96) (*SI Appendix*, Table S4).

## Discussion

### Empathy, resemblance and relatedness: the anthropomorphic stimuli hypothesis

The ability to understand others’ feelings through empathy is crucial for successful social interactions between humans^19,20^. Our predispositions for empathy are partly determined by our genes^21^ and, in all likelihood, this prosociality driver has been selected during the evolution of our species, in facilitating coordination and cooperation between individuals^1,13,22^. The extension of our empathic sensitivity toward other living beings remains nevertheless an issue poorly explored from an evolutionary perspective.

Our results show that our ability to empathize considerably fluctuates from one species to another, and that its magnitude mostly depends on the phylogenetic distance that separates them from us. Although relatedness and resemblance (*sensu* overall similarity) refer to different concepts, they empirically tend to be correlated. In an anthropocentric frame of reference, it can therefore be postulated that relatedness (here expressed as the divergence time) correspond to a rough holistic approximation of the total amount of shared external traits inherited from our common ancestor (synapomorphies), as retrospectively, they are expected to decrease relatively gradually over a long period of divergence.

Based on our results, we here hypothesize that our ability, real or supposed, to connect emotionally with other organisms would mostly depend on the quantity of external features that can intuitively be perceived as homologous to those of humans. The closer a species is to us phylogenetically, the more we would perceive such signals (and treat them as *anthropomorphic stimuli)*, and the more inclined we would be to adopt a human to human-like empathic attitude toward it. Intuitively, the correlation could have been expected but actually the assumption was not so obvious as it seemed. Indeed, in the phylogenetic thinking, overall similarity (the external features we do perceive) is not phylogenetic relatedness (ex. the coelacanth is perceived more similar to the trout than to us, whereas it is more closely related to us than to the trout). It is interesting to note that, in spite of this difficulty, overall external similarity as it generates an anthropomorphic stimuli, is still globally correlated to phylogenetic relatedness.

Consistently with the anthropomorphic stimuli hypothesis, the overall linear correlation between empathic perceptions and phylogenetic divergence time suggests differences of degree, and not differences in kinds, in the perceptions we have of the different organisms. Indeed, our data do not show any break in our empathic perceptions that would explain the customary ethical stances opposing the intrinsic values of humans *versus* other organisms (ex. Abrahamic religions, humanism), tetrapods *vs* “fishes” (ex. pesco-vegetarianism), animals *vs* plants (ex. antispecism, veganism) or vertebrates *vs* non-vertebrates (ex. various system of regulations promoting animal welfare). In such representations, values manage relationships between us and other species in terms of oppositions, while our senses perceive a gradient of shared features between us and other species. Overall, these results suggest that raters recorded what is shared in the realm of perceptions, rather than mobilizing oppositions in the realm of ethical values. Likewise, we noticed that despite the fact that some rater’s traits (such as opinions on the value of an animal’s life comparatively to those of a human) can have an effect on empathy scores, their values remain overall strongly correlated with the time of divergence.

Interestingly, the retrospective inflexion (estimated at 611.1 Mya, 95% CI: 518–703 Mya) and the stagnation of the empathic perceptions curve coincides with the transition from gnathostomes (jawed vertebrates) to non-gnathostomes (lampreys and all the others clades whose divergence from us is equal or superior to 615 Mya). Nevertheless, such an estimate is unprecise and should be considered with caution. The stagnation of our perceptions might also correspond to the prebilaterian organisms (in our dataset, all the sampled clades that have diverged from our lineage 824 Mya or earlier). Indeed, bilaterians, to which we belong, are characterized by a bilateral symmetry, with a ventrodorsal and an anteroposterior axis. Most often, they are mobile and have a head (concentration of the mouth, sense organs, and nerve ganglia at the front end). In contrast, clades having diverged from our lineage prior to bilaterians (cnidarians, fungi and plants in the present study) are lacking all these external traits and are most often sessiles. The plesiomorphic anatomical organization of these *neither heads nor tails* organisms can be destabilizing from a perceptual point of view: It is almost impossible to spontaneously establish structural or behavioral homologies connecting them to us, likely reducing our empathic projection ability to its minimum. Accordingly, several bilaterian organisms having secondarily lost externally visible bilateral symmetry (echinoderms) or undergone spectacular changes of their anatomical organization (tunicates and bivalves) present minimal empathic scores among bilaterians (their empathic scores are actually equivalent to those attributed to non-bilaterian organisms, what may have contributed to the shift of the inflection point of the curve toward a more recent time). Among the macroscopic organisms present in our sampling, such a low level of empathy is interpreted as the most basic anthropomorphic signal, and may correspond with the recognition of an entity as a living being. Overall these results suggest that humans are relatively indifferent to organisms that do not show obvious signs of antero-posterior and dorso-ventral differentiations.

### Shift between empathy and compassion

The extension of altruistic intentions (eg. sympathic or compassionate behaviours) to other organisms remains enigmatic from an evolutionary perspective, especially if we consider the latter as potential competitors, predators or as a valuable food resource for our species^23^.

Our data shows that empathy and compassion scores are significantly correlated to each other, and that both decrease with divergence time. These results were relatively expected as empathy is known to promote compassionate responses, although the neuronal networks recruited by each of these mental states have been shown to be distinct^19^.

Nevertheless, the trends obtained in these two analyses differ in several ways (Fig. 4):

(i) the correlation with divergence time is less pronounced for compassion scores than for empathy scores, and the decrease in scores with divergence time is slower for compassion than for empathy; (ii) the retrospective inflexion and the stagnation of the compassion scores curve seems to occur more recently than for the empathy scores (564.9 Mya for compassion scores versus 611.1 Mya for empathic scores, *SI Appendix*, Figs. S1 *and* S2); (iii) recorded response times are significantly higher for the compassion test, suggesting here a greater hesitation from the raters, but the differences in response time for each type of question is remarkably steady and independent from the phylogenetic distance between two species (Fig. 3B); (iv) some features of the evaluators (e.g., diet) have a confounding effect on the probability of choosing the closest phylogenetically related species that is more pronounced for compassion scores than for empathy scores (Fig. 3A, *SI Appendix*, Tables S2 and S3); and finally, v) for few taxa only, the decisions made by the raters in the compassion questionnaire are strikingly dissociated from the empathic perceptions they felt (Fig. 4). Indeed, although empathic scores attributed to tick and oak tree are relatively well corresponding to those obtained for the others protostomians and plants, respectively, their compassion scores are notably disconnected from those attributed to their relatives (strikingly lower for the tick and higher for the oak). The compassion score given to the tick is actually so low (well below the plateau formed by all the other low compassion score species) that it could be tempting to consider this result as a sharp expression of antipathy rather than as a mere lack of compassion. The strong aversion to parasitic species is not surprising given the threat they represent, and might explain the observed dissociation between empathic perceptions and compassionate responses. However, this trivial interpretation is counterbalanced by the fact that another potentially threatening species, the great white shark, reached compassion score relatively high in comparison with both its empathic score and phylogenetic distance from humans. The high compassion score for the oak tree also represents an outlier difficult to interpret. The imposing size of trees, their slow growth and long lifespan, their upright shape vaguely reminiscent of a human silhouette or their symbolic weight (which might itself results from the biological properties previously mentioned) are among the possible factors explaining the strong affective bond with trees, despite the obvious difficulties of being in empathy with a plant. Interestingly, the oak and the white shark have in common to be large sizes organisms, a trait that has been shown to positively influence our taxonomic preferences within vertebrates^8,9^.

Overall, these results led us to consider that compassionate responses, although strongly structured by intuitive empathic perceptions, nevertheless tend to be modulated by the personal ethical inclination toward non-human organisms and by the knowledge we have acquired about each species. Therefore, the compassion score as developed in our study is likely not a strict measure of the intensity of our spontaneous compassionate impulse. Whereas the empathic questionnaire is morally and affectively neutral (impression to better understand the emotions of one of the species presented in each pair), the compassion questionnaire was designed to involve emotionally the raters as much as possible. It is dilemmatic and virtually engaging their responsibility, since choosing to save one individual of the pair indirectly implies the sacrifice of the remaining one. At the end of the test, several raters have even spontaneously informed us about the discomfort perceived during certain choices they had to make. For these reasons, it would likely be more accurate to consider the compassion score as a complex expression of spontaneous emotional responses (the death of which of these two individuals would *affect me* the most?) mitigated by ethical considerations (which one *deserves* the most to survive?). Nevertheless, despite the probable intervention of reason in this rebalancing, it is remarkable to note that compassion scores remain closely linked to our spontaneous empathic perceptions and our phylogenetic proximity with a given organism.

### Sympathy beyond the confines of man

Phylogenetic distance separating us from a given organism is a key parameter to explain our predisposition to connect emotionally with the different life forms. This finding supports the hypothesis of a significant biological component at the origin of our taxonomic preferences, although additional studies involving non-occidental raters (ex. hunter-gatherer or pastoral societies) would be necessary to ensure this trend can be generalized to the whole humankind. The fluctuations of our affective preferences are likely corresponding to the amount of traits shared with humans, gradually acquired over the long term evolution of the lineage leading to us, and that are involved in the intraspecific recognition of our fellow human beings. To some extent, such anthropomorphic stimuli induced by other organisms could therefore mobilize a cognitive circuitry that is usually at work in human relationships. The emotional reactions and prosocial behaviors they may promote would therefore be all the stronger as the species is close to us, as it shares with us more of these traits.

This phenomenon evokes similarities with the interspecific behavioral diversions episodically reported in other vertebrates providing parental care. Incidental cases of interspecific adoption - most often between relatively closely related species - are well documented in mammals and birds^24,25^. Some birds, such as the cuckoos (*Cucculus* sp.) have even turned these behavioral flaws at their advantage, through a successful brood parasitic way of life, in forcing the adoption of their offspring by parents from another species^26^. However, it may be reductive to consider the derivation of human prosocial traits from the sole perspective of a selective disadvantage. Our interactions with other organisms are highly diversified and little is known about the real impact our emotions toward them may have had on the human evolution. Our empathic skills may have for instance offered to early hominids the advantage to better anticipate the reactions of wild mammals, either to facilitate their hunt or to assess instantaneously and individually their mood and the danger they may represent. Likewise, our compassionate impulses may have pushed our ancestors to rescue injured or hungry animals, or to adopt young orphaned animals. To what extent could such altruistic interactions between humans and animals have preceded and contributed to the emergence and the long-run development of the multiple domestication episodes remains unknown. What do we know, for instance, about the cognitive predispositions and the motivations that may have allowed humans to make the dog – proverbially presented as our best friend – the very first of the domesticated species?

## Methods

### Ethics statement

All experiments were performed in accordance with relevant guidelines and regulations. The French National Commission on Informatics and Liberty approved protocols for this study (CNIL number 2-19061). All participants were informed of the subject of the study (perception of biological alterity) and the protocol for processing personal data on the first page of the website, and access to experiments was conditional on an explicit informed consent to participate. Data were collected and analyzed anonymously.

### Photographic stimuli

Pictures of a diversified set of 52 macroscopic eukaryote species have been selected (47 animal species - including *Homo sapiens*, four plants and one fungi). Although any species sampling involved in a comparative study of the diversity of life on a large scale inevitably has an arbitrary component, our sampling has been developed in order:

1. To optimize the representativeness in terms of phylogenetic diversity, which translates here into the representativeness in terms of temporal divergence from humans, given the hypothesis to be tested: In that respect, most of the clades connecting at different level of the tree of life and that are placed as sister clades of the lineage leading to humans are represented^27,28^. Nevertheless, microscopic organisms have been excluded despite the fact they make up a considerable part of the biodiversity, because we considered them to be beyond our common sensory reach. In total, and excluding *H. sapiens*, our sampling represent 24 clades that diverged from the lineage leading to man at different times, from our sister clade (chimpanzee, 6.7 Mya) to the very distantly related plants clade (1496 Mya)^27^ (Fig. 2., *SI Appendix*, Table S1).
2. To optimize the representativeness of the phenotypic and phylogenetic diversity among each of these clades: Most of them are represented by several species that have been selected to be highly divergent from each other (i.e. intra-clade divergence time values are always ≥45 million years)^27^. Species selected for a given clade can therefore be considered as different taxonomic samples (i.e. replicates) in order to measure the variability of our empathic reactions for a given divergence time value. Eleven poorly diversified clades (most often very closely related to humans) are represented by a single species (ex. Panina, Gorillini, Ponginae) whereas up to eight highly divergent species have been selected in order to take into account polymorphism of hyperdiversified lineages such as protostomians. For this particular clade, we have for instance selected three very divergent mollusks (a snail, a cuttlefish and a scallop), one annelid (an earthworm) and four very different arthropods (a beetle, a shrimp, a spider and a tick). Given that domestic species have been transformed by human selection, they have been excluded from the sampling because it is likely that their evolution have been directionally driven by our empathic or aesthetical preferences. As far as possible, species overrepresented in the media and entertainment (e. g. bottlenose dolphin) have been avoided or replaced by closely related species that are less popular (e.g. beluga whale) (*SI Appendix*, Table S1).
3. To take into account the variability among each species: For each species, four distinct photographs of distinct living individuals have been selected from online open sources in order to represent phenotypic variation of living individuals and minimize the enhancement bias specific to each shot (N total = 208 photos). Only photographs representing adult individuals were selected, as it has been shown that in mammals juvenile traits can positively influence our empathic perceptions^11,12^. For humans, two women and two men representing four distinct ethnic phenotypes have been selected.

### Procedure

An online application was generated to present random pairs of photographs of different species. No information on the photographed individuals was given to the participants, who could therefore only base their choice on images. For each pair, the rater was instructed to click on the photograph corresponding to the answer to one of the two specific questions randomly chosen for each rater (Fig. 1). The position of the photograph on the screen (left or right) was randomly ascribed for each pair and for each rater. Each rater had 22 distinct pairs of photographs to assess, randomly drawn from the set of 52 species, with the constraint that for each pair, the two species were drawn from distinct clades, and that no species is seen more than once. Three pairs, randomly chosen from among those previously viewed (excluding the last four pairs already seen), were presented again at the end to estimate judgment reliability.

### Raters

A total of 3509 raters participated, between November and December 2018. For each rater, the following general information was collected: sex, year and month of birth, and nationality. In addition, each rater provided information on his *knowledge on biodiversity* (poor, average, good, advanced), *type of diet* (omnivorous, vegetarian (fish allowed), strictly vegetarian, vegan), *type of pet owned* (yes or no for 5 categories: mammal, fish, reptile, bird, arthropod), *opinion on hunting and fishing* (practicing or supporting, against, indifferent), and *opinion on the value of animal life* (none, low, some but lower than human’s, equal to human’s, higher than human’s), see *SI Appendix, Methods* for further details and the french original version. The following conservative selection on raters was applied. First, to reduce cultural heterogeneity, only raters from an European nationality were considered. Second, unreliable raters (i.e., with more than one incorrect answer during the test of judgment reliability), non-adult raters (lower than 18 years old), or raters with incomplete data were removed. Finally, evaluation of pair of photographs taking less than 200 ms or more than 7 min were discarded. A total of 2347 raters were retained in the final sample (1134 for the empathy test and 1213 for the compassion test), corresponding to 1434 females (mean age: 37.27 + /− 0.34 years old, range: 18.1–78.5) and 916 males (mean age: 38.04 + /− 0.43 years old, range: 18.2–81.2). Each photograph was seen, on average, by 501.3 raters (range: 474–529).

### Statistics

The aim was to examine the influence of the phylogenetic divergence time relatively to the human species on answers to empathy driven or compassion driven questions. Distinct analyses were performed for each question. Logistic regressions were used to analyze raters’ decisions. The binary response variable corresponded to being chosen or not for the focal species (arbitrarily the species presented at the left position) during the presentation of each pair. Species and raters were considered random samples from a larger population of interest and were thus random-effect variables. Therefore, generalized linear mixed models with a binomial error structure were used. For each choice made by a rater, the difference between the phylogenetic divergence time with humans of the focal and the non-focal species was calculated, as provided by timetree.org^27^. The value of this difference was integrated into the model as the main variable of interest (*Test*). To control for potential confounding effects, variables concerning the raters’ characteristics were also included in the model (after pooling some categories poorly represented) as interaction terms with the variable of interest. These confounding variables were the rater’s sex (qualitative: male, female), age (quantitative, centered), knowledge on biodiversity (qualitative: minimum, average, fair, good), type of diet (qualitative: omnivorous, pesco-vegetarian, vegetarian), number of types of pet owned (quantitative, centered), opinion on hunting and fishing (qualitative: supporting, indifferent, against), and opinion on the value of animal life relatively to humans (qualitative: lower, equal, higher). The significance of each independent variable was calculated by removing it from the full model and comparing the resulting variation in deviance using a Chi square test. For each question, a score was computed for each species, computed as the number of time the species was chosen, divided by the number of time it was presented to raters. These computations were done using the lme4 package^29^ on R 3.5.1 software (R Core Team 2018). The inflexion point (IP) of each Time-Scores curves was defined as the time when the slope of the fitted line changed. It was estimated by fitting a broken line, corresponding to four parameters: IP, slopes of the line before and after IP, and coordinate at t = 0. These parameters were estimated by minimizing the sum of the squares of the residuals, using a genetic optimization algorithm as implemented in the R package rgenoud, version 5.8-3.0^30^. Confidence interval for IP was calculated by bootstrap, using at least 5000 resamples with replacement.

## Supplementary information

Supplementary Information
