# Supplementary material for: Empathy and compassion toward other species decrease with evolutionary divergence time
Source: Sci Rep. 2019 Dec 20;9:19555. doi: 10.1038/s41598-019-56006-9 (PMC6925286; doi:10.1038/s41598-019-56006-9)
Supplement: Supplementary file 1 — Supplementary Information [file 41598_2019_56006_MOESM1_ESM.pdf]

Supplementary Information

Article title: Empathy and compassion toward other species decrease with evolutionary divergence time.

Authors : Aurélien Miralles[1*], Michel Raymond[2]†, Guillaume Lecointre[1]†

Affiliation : [1]Institut de Systématique, Evolution, Biodiversité, (UMR 7205 Muséum national d'Histoire naturelle, CNRS UPMC EPHE, Sorbonne Universités), CP30, 25 rue Cuvier 75005 Paris, France; [2]ISEM, Univ Montpellier, CNRS, EPHE, IRD, Montpellier, France

- Supplementary text Method S1
- Figures S1 to S2
- Tables S1 to S4

Methods S1. Questionnaire (original questions in French in blue)

Personal informations questionary:

What is your sex ? / *Quel est votre sexe ?*
□ Female / *Femme*
□ Male / *Homme*

What is your year and month of birth ?/ *Quels sont votre année et mois de naissance ?*

What is your nationality ? / *Quelle est votre nationalité ?*

What is your knowledge of biodiversity (Fauna, Flora...) ? / *Quelles sont vos connaissances sur la biodiversité (Faune, Flore..) :*
□ rather minimal / *Plutôt minimes.*
□ in the average / *Dans la moyenne.*
□ Rather good (It interests me) / *Plutôt bonnes (ca m'intéresse).*
□ Advanced (training in biology, naturalistic activities…) / *Poussées (formation en biologie, activités naturalistes…).*

What is your diet ? / *Quel est votre régime alimentaire ?*
□ Omnivorous (I eat everything) / *Omnivore (je mange de tout)*
□ Pesco-vegetarian (no meat, but I can eat fish) / *Pesco-végétarien (pas de viande, mais je m'autorise le poisson)*
□ Vegetarian (no meat nor fish) / *Végétarien (ni viande ni poisson)*
□ Vegetalian/vegan (no animal products at all) / *Végétalien/Vegan (aucun produit d'origine animale)*

What are your pets ? / **Quels sont vos animaux de compagnie ?**
Yes No
□ □ Dog, cat, rodent or any others fur animal / *Chien, chat, rongeur ou tout autre animal à poils*
□ □ Fish / *Poissons*
□ □ Snake, turtle, lizard, amphibian / *Serpent, tortue, lézard, amphibien*
□ □ Insect, spider, crustacean… / *Insecte, araignée, crustacé…*
□ □ Bird / *Oiseau*

What is your position on hunting and fishing: / *Quelles est votre position sur la chasse et la pêche:*
□ I practice (or support) recreational hunting and fishing / *Je pratique (ou soutient) la chasse et de la pêche de loisir*
□ I am against recreational hunting and fishing / *Je suis contre la chasse et la pêche de loisir*
□ Neither one or the other / *ni l'un ni l'autre*

In my opinion, the life of an animal…. / *Selon moi, la vie d'un animal…*
□ has no value / *n'a aucune valeur.*
□ has little value / *a peu de valeur.*
□ has value, but that of a human being is superior to it / *a de la valeur, mais celle d'un être humain lui est supérieure.*
□ is equal to that of a human being / *est égale à celle d'un être humain.*
□ is superior to that of a human being / *est supérieure à celle d'un être humain.*

Photographic questionnaire : either to assess empathy (A) or compassion (B)

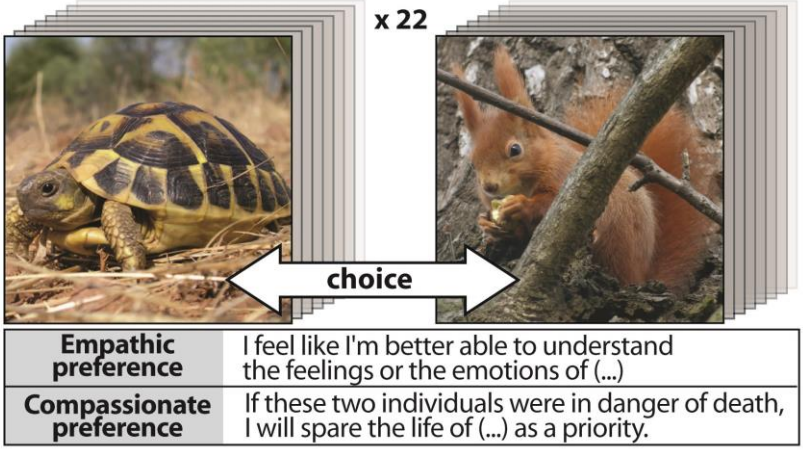

a) J'ai l'impression de mieux reconnaitre / comprendre les sentiments / émotions de (…)
b) Si ces deux individus étaient en danger de mort, j'épargnerais en priorité la vie de (…)

Figure S1. (A) Empathy and (B) compassion scores attributed to each organism as a function of divergence time between them and humans. The scores correspond to the probability that a given species is chosen from a pair of species that includes it and another randomly selected. The graphs on the right side show the mean scores obtained for each clade.

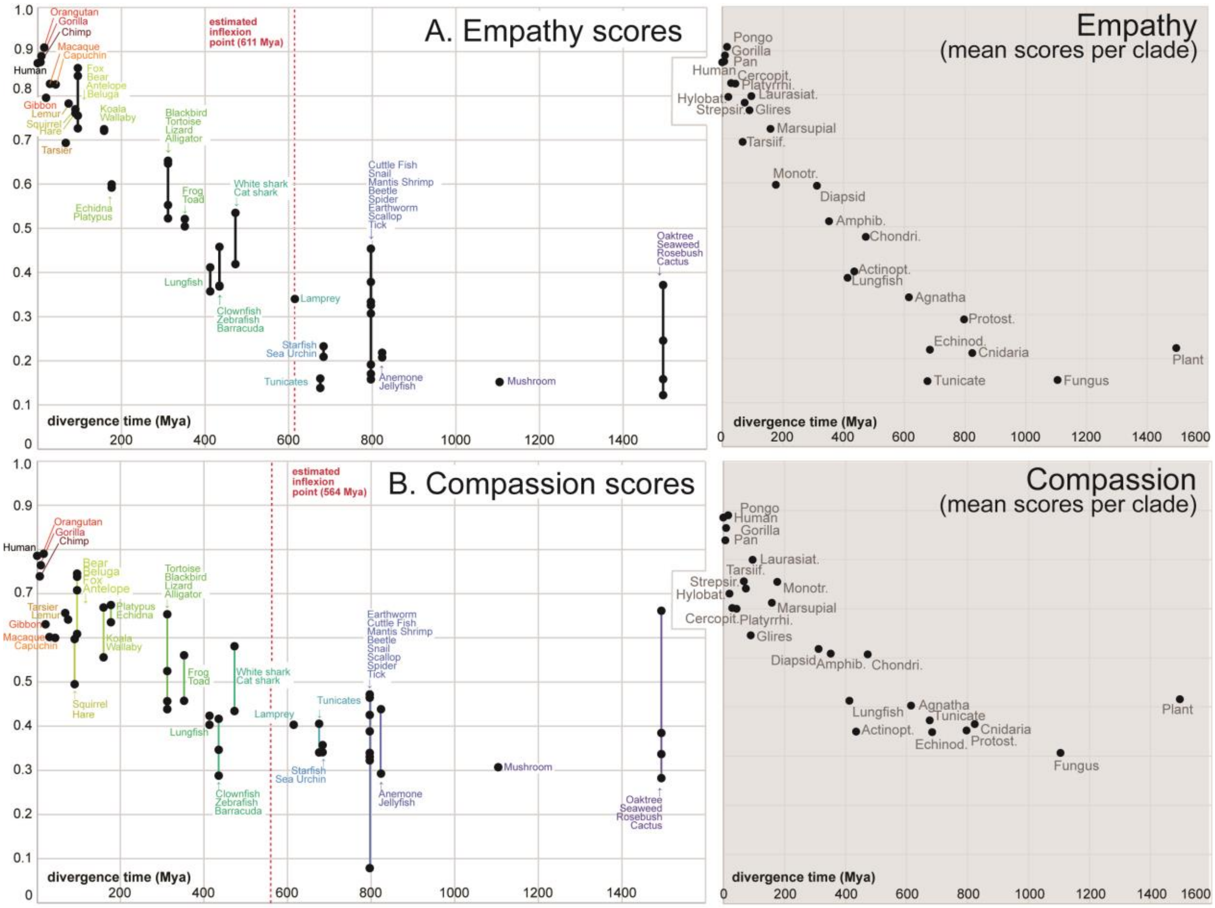

Figure S2. Inflexion points estimated for the empathy score-divergence time and compassion-scores – divergence time curves. The confidence intervals were calculated by bootstrap, using at least 5000 resamples with replacement.

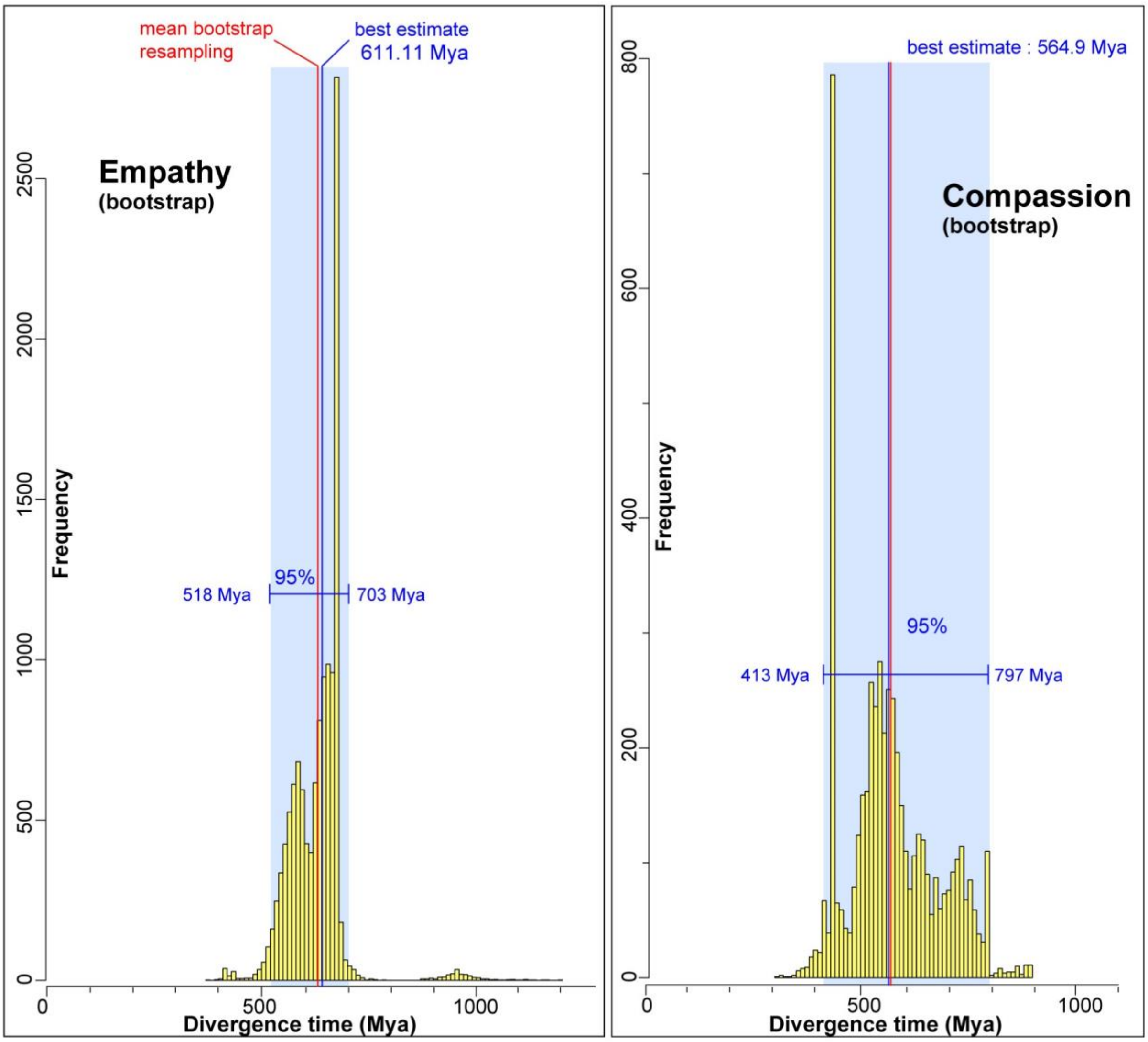

Table S1. Taxonomic sampling, divergence time between humans and each species (estimated divergence time from Kumar *et al*. (2017) and empathy and compassion scores. The scores correspond to the probability that a given species is chosen from a pair of species that includes it and another randomly selected.

**Clade (N species sampled)**	**Divergence time (My)**	**Species**	**Common name**	**Empathy scores**	**Compassion score**
**Hominina (1)**	0	*Homo sapiens*	Human	0.874	0.785
**Panina (1)**	6,65	*Pan troglodytes*	Chimpanzee	0.876	0.739
**Gorillini (1)**	9,06	*Gorilla gorilla*	Gorilla	0.890	0.764
**Ponginae (1)**	15,76	*Pongo sp.*	Orangutan	0.909	0.790
**Hylobatidae (1)**	20,19	*Hylobates lar*	Lar Gibbon	0.795	0.630
**Cercopithecidae (1)**	29,4	*Macaca fuscata*	Japanese macaque	0.827	0.602
**Platyrrhini (1)**	43,2	*Cebus capucinus*	Capuchin	0.825	0.600
**Tarsiiformes (1)**	67,1	*Tarsius sp.*	Tarsier	0.693	0.656
**Strepsirrhini (1)**	74	*Lemur catta*	Ring-tailed lemur	0.782	0.641
**Glires (2)**	90	*Lepus sp.*	Hare	0.760	0.495
—	—	*Sciurus vulgaris*	Red Squirrel	0.769	0.597
**Laurasiatheria (4)**	96	*Antidorcas marsupialis*	Springbok antelope	0.726	0.608
—	—	*Ursus arctos*	Brown bear	0.845	0.745
—	—	*Delphinapterus leucas*	Beluga whale	0.755	0.738
—	—	*Vulpes vulpes*	Red Fox	0.863	0.707
**Marsupialia (2)**	159	*Phascolarctos cinereus*	Koala	0.724	0.669
—	—	*Macropus rufogriseus*	Benett’s wallaby	0.720	0.556
**Monotremata (2)**	177	*Tachyglossus sp.*	Echidna	0.599	0.635
—	—	*Ornithorhynchus anatinus*	Platypus	0.592	0.674
**Diapsida (4)**	312	*Alligator mississippiensis*	Mississipi alligator	0.522	0.438
—	—	*Turdus merula*	Common blackbird	0.653	0.525
—	—	*Teira duguesi*	Madeiran wall Lizard	0.552	0.456
—	—	*Testudo hermanni*	Hermann’s tortoise	0.646	0.653
**Amphibia (2)**	352	*Rhinella marina*	Cane toad	0.504	0.457
—	—	*Hyla arborea*	European tree frog	0.521	0.560
**Dipnoi (2)**	413	*Neoceratodus forsteri*	Queensland Lungfish	0.357	0.423
—	—	*Protopterus amphibius*	Gilled Lungfish	0.411	0.403
**Actinopterygii (3)**	435	*Sphyraena sp.*	Barracuda	0.368	0.346
—	—	*Amphiprion ocellaris*	Common clownfish	0.458	0.417
—	—	*Danio rerio*	Zebrafish	0.370	0.288
**Chondrichthyes (2)**	473	*Scyliorhinus canicula*	Small-spotted catshark	0.419	0.434
—	—	*Carcharodon carcharias*	Great white shark	0.535	0.581
**Agnatha (1)**	615	*Petromyzon marinus*	Sea lamprey	0.339	0.403
**Tunicata (2)**	676	*Clavelina caerulea*	Blue sea squirt	0.160	0.405
—	—	*Ciona edwardsi*	Yellow sea squirt	0.139	0.340
**Echinodermata (2)**	684	*Pisaster ochraceus*	Ochre starfish	0.232	0.357
—	—	*Paracentrotus lividus*	Purple sea Urchin	0.209	0.341
**Protostomia (8)**	797	*Timarcha sp.*	Bloody-nosed beetle	0.326	0.388
—	—	*Sepia officinalis*	Common cuttlefish	0.453	0.464
—	—	*Lumbricus sp.*	Earthworm	0.191	0.471
—	—	*Odontodactylus scyllarus*	Peacock mantis shrimp	0.333	0.425
—	—	*Pecten maximus*	Great scallop	0.170	0.330
—	—	*Helix pomatia*	Burgundy snail	0.378	0.339
—	—	*Araneus diadematus*	Diadem spider	0.307	0.322
—	—	Ixodida *gen. sp.*	Tick	0.158	0.078
**Cnidaria (2)**	824	*Actinostola sp.*	Sea Anemone	0.218	0.438
—	—	*Chrysaora sp.*	Jelly Fish	0.207	0.292
**Fungi (1)**	1105	*Boletus edulis*	Cep mushroom	0.152	0.307
**Plantae (3)**	1496	*Echinocactus sp.*	Barrel cactus	0.158	0.282
—	—	*Quercus sp.*	Oak tree	0.371	0.661
—	—	*Fucus vesiculosus*	Rockweed	0.122	0.384
—	—	*Rosa sp.*	Rosebush	0.246	0.336

Reference : Kumar S, Stecher G, Suleski M, Hedges SB (2017) TimeTree: A Resource for Timelines, Timetrees, and Divergence Times. *Mol Biol Evol* 34:1812–1819. doi: 10.1093/molbev/msx116

Table S2. Effect of each variable on the choice made by raters; for both types of questions. Bold characters indicate significant ($P < 0.05$) values.

	Empathy driven question			Compassion driven question		
**Fixed effects**	**Chisq**	**Df**	**P-value**	**Chisq**	**Df**	**P-value**
Test	921.7	1	**$<2.2 \times 10^{-16}$**	600.9	1	**$<2.2 \times 10^{-16}$**
Interaction with:						
Sex	5.24	1	**0.022**	0.783	1	0.376
Age	11.4	1	**0.001**	15.390	1	**8.743×10^{-05}**
Diet	1.24	2	0.536	21.475	2	**2.171×10^{-05}**
Knowledge	19.3	3	**0.000**	11.280	3	**0.010**
Fishing/Hunting	8.48	2	**0.014**	13.690	2	**0.001**
Life value	48.7	2	**2.7×10^{-11}**	92.044	2	**$<2.2 \times 10^{-16}$**
Pet	0.04	1	0.837	5.814	1	**0.016**

Table S3. Effect of participants sociological traits on empathy and compassion scores.

**A.**	**Empathy driven question**				**Compassion driven question**			
**Fixed effects**	**Estimate**	**SE**	**z-value**	**Pr(>|z|)**	**Estimate**	**SE**	**z-value**	**Pr(>|z|)**
(Intercept)	0.1111	0.158	0.705	0.481	0.048	0.103	0.468	0.639
Test	2.536	0.187	13.606	**$<2x10^{-16}$**	0.629	0.126	5.003	**$5.6x10^{-7}$**
Interaction with:								
Sex (male)	0.240	0.105	2.288	**0.022**	0.063	0.072	0.885	0.376
Age	-0.0129	0.004	-3.373	**0.001**	-0.011	0.003	-3.923	**$8.7x10^{-5}$**
Diet pesco	-0.0109	0.192	-0.056	0.955	0.261	0.153	1.706	0.088
Diet : vege	-0.196	0.176	-1.113	0.266	0.610	0.136	4.489	**$7.2x10^{-6}$**
Knowledge : good	-0.123	0.185	-0.662	0.508	-0.243	0.131	-1.852	0.064
Knowledge : average	0.031	0.177	0.177	0.859	-0.029	0.129	-0.222	0.824
Knowledge : advanced	0.397	0.171	2.319	**0.021**	0.052	0.124	0.423	0.672
Fishing/hunting : cons	0.180	0.109	1.654	0.098	0.185	0.077	2.405	**0.016**
Fishing/hunting : pros	-0.353	0.182	-1.934	0.053	-0.262	0.119	-2.194	**0.028**
Life : human >animal	0.610	0.113	5.406	**$6.4x10^{-8}$**	0.633	0.075	8.389	**$<2x10^{-16}$**
Life : animal > human	-0.685	0.195	-3.517	**$4.4x10^{-3}$**	-0.471	0.147	-3.193	**0.001**
Pets	-0.012	0.054	-0.206	0.837	0.096	0.040	2.411	**0.016**

Table S4. Analysis of deviance table for the time of response. The type of question (empathy or compassion driven) and the absolute phylogenetic distance between the species depicted in the pairs are the independent variables. The test refers to a type II Wald chisquare test.

	**Chisq**	**Df**	**Pr(>Chisq)**
Type of question	79.25	1	**$<2x10^{-16}$**
Phylogenetic distance	180.62	1	**$<2x10^{-16}$**
